# miR-21 expression and clinical outcome in locally advanced pancreatic cancer: exploratory analysis of the pancreatic cancer Erbitux, radiotherapy and UFT (PERU) trial

**DOI:** 10.18632/oncotarget.7208

**Published:** 2016-02-05

**Authors:** Khurum Khan, David Cunningham, Clare Peckitt, Sarah Barton, Diana Tait, Maria Hawkins, David Watkins, Naureen Starling, Sheela Rao, Ruwaida Begum, Janet Thomas, Jacqui Oates, Vincenza Guzzardo, Matteo Fassan, Chiara Braconi, Ian Chau

**Affiliations:** ^1^ Gastrointestinal Unit, The Royal Marsden NHS Foundation Trust, Sutton, UK; ^2^ CRUK/MRC Oxford Institute for Radiation Oncology, Gray Laboratories, University of Oxford, Oxford, UK; ^3^ Department of Medicine, University of Padua, Padua, IT; ^4^ Division of Cancer Therapeutics, The Institute of Cancer Research, Sutton, UK

**Keywords:** pancreatic cancer, microRNA, miR-21, chemo-radiotherapy, cetuximab

## Abstract

**Background:**

Locally advanced pancreatic cancer (LAPC) is associated with high mortality, and biomarker-driven treatment approach is currently lacking. This study evaluated safety and efficacy of a combination approach of chemotherapy followed by chemo-radiotherapy (CRT) +/− cetuximab, and the prognostic role of miR-21 in patients with LAPC treated with a multimodality approach.

**Patients and Methods:**

This was a randomised phase II trial in which patients with inoperable LAPC were offered gemcitabine and capecitabine (GEM-CAP) for 16 weeks. Patients with stable disease or response after GEM-CAP were randomised to capecitabine or UFT plus radiotherapy (RT) (A), or capecitabine or UFT plus cetuximab plus RT (B). The primary outcome of the study was overall survival (OS). Clinical outcome was compared according to baseline circulating miR-21 levels.

**Results:**

17 patients were enrolled and treated with GEM-CAP, with 13 patients achieving disease control and being randomised to arms A (n:7) and B (n:6). After a median follow-up of 61.2 months, median progression free survival (PFS) was 10.4 months and 12.7 months, median OS was 15.8 months and 22.0 months in arms A and B respectively (*p* > 0.05). Patients with high baseline plasma miR-21 had worse PFS (3.5 *vs*. 12.7 months; p:0.032) and OS (5.1 *vs* 15.3 months; p:0.5) compared to patients with low miR-21. Circulating miR-21 levels reflected miR-21 expression within the tissues.

**Conclusions:**

Addition of Cetuximab to CRT following induction chemotherapy did not improve survival. High miR-21 baseline plasma expression was associated with poor clinical outcome in LAPC patients treated with induction chemotherapy followed by chemo-radiotherapy.

## INTRODUCTION

Despite the recent advancements in diagnosis and management of solid malignancies, pancreatic ductal adenocarcinoma (PDAC) remains a highly lethal disease [1]. Majority of patients present with locally advanced unresectable (40-50%) or metastatic disease (40%) [2]. Management of Locally advanced Pancreatic Cancer (LAPC) remains largely under-researched with lack of evidence both in terms of optimal treatment approach and biomarkers that could inform such an approach [3]. Although previously conducted studies failed to demonstrate any definite survival advantage of chemo-radiotherapy (CRT) over chemotherapy (CT) alone [4, 5], retrospective analysis of 4 phase II-III studies demonstrated that patients without disease progression after 3 months of CT, followed by CRT had a longer survival than those continuing on CT alone [6]. A pooled meta-analysis of SAKK and UK studies showed clinical activity for gemcitabine and capecitabine (GEM-CAP) combination [7, 8], suggesting this could be considered a useful neo-adjuvant chemotherapy (NACT) approach. Recent evidence suggests that addition of epidermal growth factor receptor (EGFR) inhibition to CRT is feasible and promising in terms of efficacy [9]. Furthermore, EGFR is known to be upregulated in up to 69-95% of advanced pancreatic cancers [10, 11] and the EGFR tyrosine kinase inhibitor erlotinib combined with gemcitabine demonstrated survival benefit over gemcitabine alone [12]. Cetuximab, an anti-EGFR antibody has been safely combined with chemotherapy and radiotherapy in patients with LAPC and with other cancers [13-15]. Additionally, pre-clinical studies have demonstrated improved efficacy when gemcitabine was combined with radiotherapy in PDAC, suggesting independent radio-sensitive effects of cetuximab in this cancer [16, 17].

MicoRNAs (miRNAs) are non-coding RNAs that have been implicated in post-transcriptional regulation of gene expression. Growing evidence suggests the role of miRNAs in regulation of carcinogenesis and modulation of drug response [18, 19]. Over-expression of miR-21 is known to be associated with decreased sensitivity to gemcitabine *in vitro* and with poor clinical outcomes in retrospective clinical studies [20-23]. Furthermore, miRNAs can be detected both in tissue and plasma thus suggesting their role as potentially useful clinical biomarkers [24].

In this randomised phase II trial of patients with LAPC, we sought to evaluate an optimal radiosensitivity agent in patients who achieved disease control (DC) after neo-adjuvant GEM-CAP. The PERU trial was closed prematurely in June 2013 because the emergent data from LAP-07 study [25] failed to demonstrate any meaningful survival advantage with the use of CRT approach following first-line chemotherapy in LAPC. However, we have taken advantage of this cohort of patients by carrying out a preliminary study on the role of circulating miRNAs as prognostic biomarkers in LAPC. Despite growing evidence supporting the value of circulating miRNAs in predicting clinical outcome in cancer patients, most of the data is generated in retrospective and unselected populations, and data in prospective clinical trials are lacking. Here we report miR-21 plasma expression and its association with clinical outcomes in this prospective study of LAPC patients treated with a combination approach.

## RESULTS

### Baseline characteristics

Seventeen patients, all with de novo LAPC were enrolled and treated with NACT. Patients and tumour characteristics have been summarised in Table 1. Sixteen (93%) patients received at least 3 cycles of NACT. Median relative dose intensity of gemcitabine was 76.2%, while that of capecitabine was 89.6%. Eighty-three percent of patients received the planned dose of RT (Supplementary Table 1).

**Table 1:** Clinico-pathological characteristics of all 17 registered patients receiving NACT

	ALL *N*	(%)
***Age (years)*** Median, (range)	61.0 (47-76)	
Gender *Male* *Female*	11 6	64.7 35.3
***ECOG Performance Status*** 0 1	3 14	17.7 82.3
***Site of Primary*** Head of Pancreas Body of Pancreas	13 4	76.5 23.5
***Radiological T-stage*** T3 T4 TX	1 13 3	5.9 76.5 17.6
***Radiological N-stage*** N0 N1 NX	5 9 3	29.4 52.9 17.6

### Efficacy and safety

**Figure 1:**
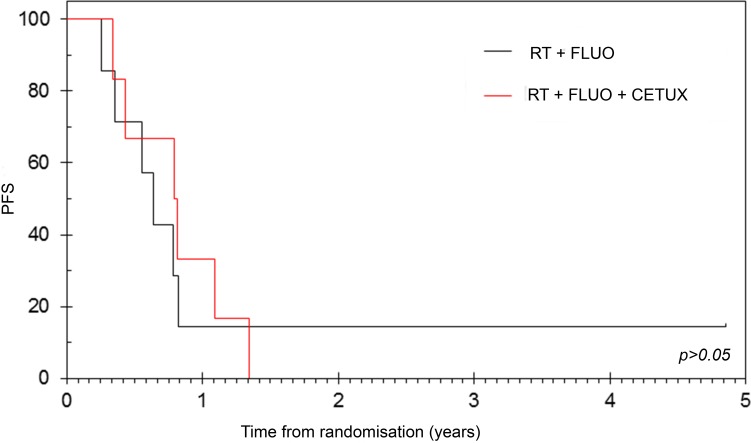
PFS according to randomisation All randomised patients (*n* = 13) who achieved disease control with neoadjuvant chemotherapy were randomised to arms A (*n* = 7) and B (*n* = 6). After a median follow-up of 61.2 months, no differences in median PFS from randomization were observed between arm A [median 7.6 months (95%CI: 5.1 - 10.3)] and arm B [median 9.5 months (95%CI: 4.1 - 15.0)]. RT = radiotherapy, FLUO: Fluoropyrimidine and CETUX = cetuximab.

Thirteen patients who achieved disease control rate (DCR) with NACT (PR = 11.8%, SD = 64.7%) were randomised to arms A (*n* = 7) and B (*n* = 6). Objective Response Rate (ORR) following CRT was 14.3% and 33.3% in groups A and B respectively (*p* > 0.05). After a median follow-up of 61.2 months, median Overall Survival (OS) from time of NACT was 15.8 (95% CI: 14.5 - 17.9) and 22.0 (95% CI: 0 - 45.5; *p* > 0.05) months while median Progression Free Survival (PFS) was found to be 10.4 (95% CI: 7.8 - 13.0) and 12.7 (95% CI: 7.3 - 18.0; *p* > 0.05) months in the two arms respectively (Figure 1). 1-year survival was 100% and 66.7% (*p* = 0.801, 95% CI: 29.1 - 100) in arms A and B respectively. OS in all registered patients (*n* = 17) was 15.3 months (95% CI: 13.0 - 17.5) and 64.7% (95% CI: 42.0 - 87.4) of the patients were alive at 1-year. NACT approach with GEM-CAP and combining cetuximab with CRT or with chemotherapy were both found to be safe (Table 2 and Supplementary Table 2).

**Table 2:** Adverse events during chemo-radiation with or without cetuximab

TOXICITY	CRT ARM A (RT+FLUO) (*n* = 6)		CRT ARM B (RT + FLUO + CETUX) (*n* = 6)	
	Grade I/II *N* (%)	Grade III/IV *N* (%)	Grade I/II *N* (%)	Grade III/IV *N* (%)
**Anaemia**	3 (50)		5 (83.3)	
**Hyperglycaemia**	5 (71.4)		4 (66.7)	
**Hyponatraemia**	5 (83.3)	1 (16.7)	4 (66.7)	
**Dry skin**	2 (33.3)		4 (66.7)	
**Constipation**			4 (66.7)	
**Rash/desquamation**			4 (66.7)	
**Thrombocytopenia**	4 (66.7)		3 (50)	
**Lethargy**	4 (66.7)		3 (50)	
**Hypocalcaemia**			3 (50)	
**Hand foot syndrome (PPE)**	3 (50)		2 (33.3)	
**Diarrhoea**	5 (83.3)		2 (33.3)	
**Anorexia**	2 (33.3)		2 (33.3)	
**Nausea**	3 (50)			
**Neutropenia**			2 (33.3)	
**Fatigue**	1 (14.2)	1 (16.7)	2 (33.3)	
**Hypokalaemia**			2 (33.3)	
**Vomiting**	2 (28.6)		1 (16.7)	
**Abdominal pain**	1 (16.7)	1 (16.7)	1 (16.7)	
**Dehydration**	2 (33.3)			
**Non Neutropenic infection**	1 (16.7)		1 (16.7)	
**Urinary frequency**			1 (16.7)	
**Allergic reaction**			1 (16.7)	
**Dizziness**	1 (16.7)		1 (16.7)	
**Dyspepsia**	2 (28.6)		1 (16.7)	
**Dyspnoea**	1 (16.7)		1 (16.7)	
**Psoriasis**			1 (16.7)	
**Acneiform rash**			1 (16.7)	
**Hyper-pigmentation**			1 (16.7)	
**Hypercalcaemia**	1 (16.7)			
**Hyperkalaemia**	1 (16.7)			
**Hypoglycaemia**	1 (16.7)			
**Hypomagnesaemia**			1 (16.7)	
**Nail changes**	1 (16.7)			
**Peripheral oedema**	1 (16.7)			
**Stomatitis**	1 (16.7)			
**Syncope**	1 (16.7)			
**Taste alteration**	1 (16.7)			

### miR-21 molecular analysis results

**Figure 2:**
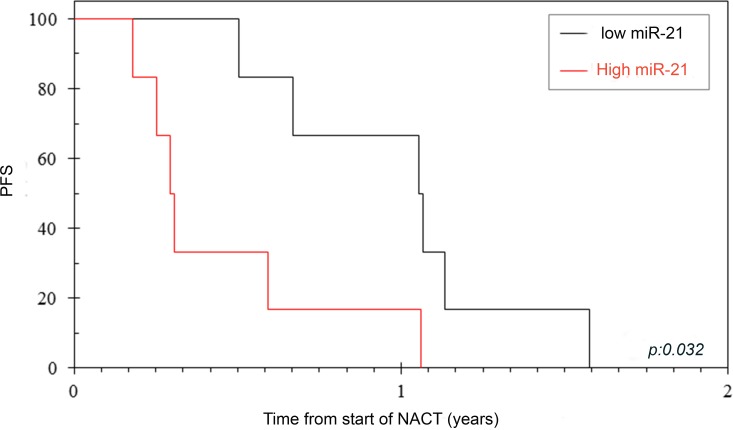
PFS according to miR-21 expression Circulating miR-21 was assessed in baseline plasma and used as a binary variable to differentiate patients with high miR-21 and low miR-21. miR-21 expression significantly correlated with PFS.

**Figure 3:**
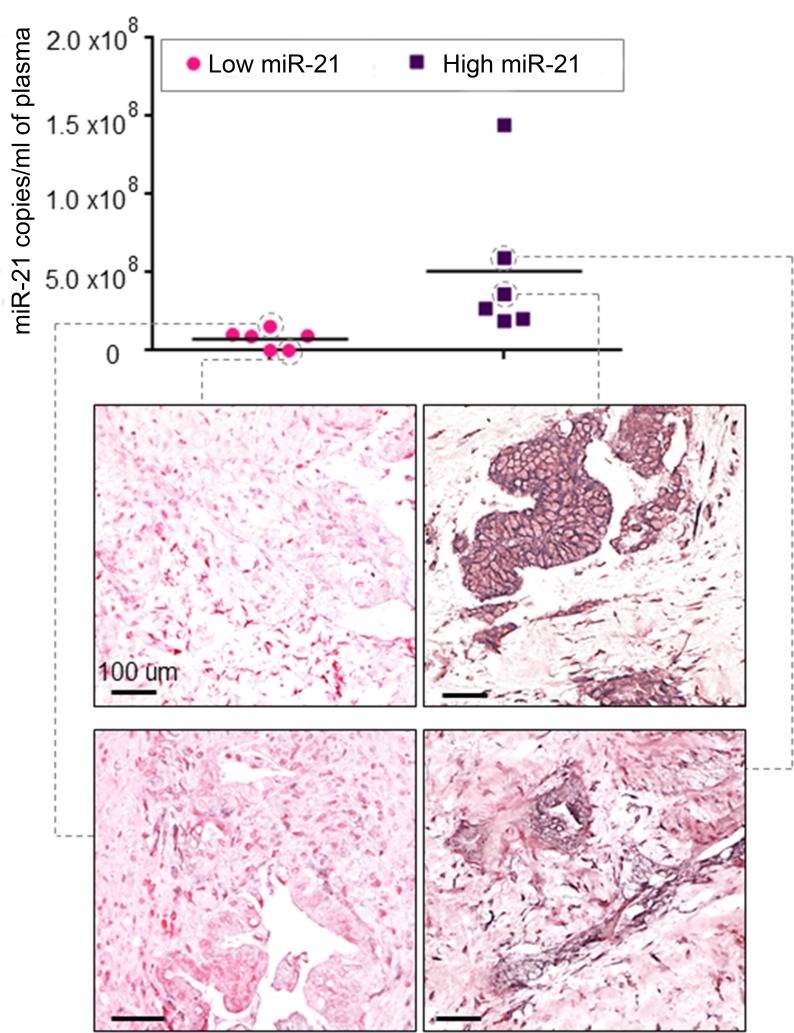
Circulating miR-21 expression reflects expression of miR-21 in tumour tissue Matched baseline plasma and Formalin-Fixed-Paraffin-Embedded tissues were analysed. Circulating miR-21 expression was quantitated by ddPCR, and tissue miR-21 expression was assessed by ISH. Patients with high circulating levels of miR-21 had tumours with high miR-21 expression in cancer cells in the tissues.

Of the 17 registered patients, plasma samples were available in 16 cases. Four cases could not be analysed because haemolysis occurred in the samples. Circulating miR-21 expression was assessed by Taqman assay in the baseline plasma sample in 12 patients, who were divided in two groups (low and high miR-21) by using the median as a cut-off. When miR-21 expression was validated by digital droplet polymerase chain reaction (ddPCR), patients were assigned to the same groups (copies of miR-21/ml of plasma ranged between 1.1E+04 and 1.4E+08). All these patients underwent NACT with GEM-CAP. Amongst these patients, 3 patients progressed on NACT, and 8 were further randomized to arm A (*n* = 4) and arm B (*n* = 4). DCR post NACT was 100% *vs*. 40% in patients with low and high miR-21 respectively (*p* = 0.06). Interestingly, all patients who progressed after NACT had high values of circulating miR-21 at baseline (Supplementary Table 3). Patients with high miR-21 plasma levels had a significantly worse PFS from registration [3.5 (95% CI: 2.8 - 4.3) *vs*. 12.7 (95% CI: 6.9 - 18.4 months); *p* = 0.032], and a non-significant trend on OS was observed (Figure 2 & Table 3). When all randomised patients were analysed, patients with low miR-21 were found to have better DCR at 6 weeks of chemotherapy [100% (5/5) *vs*. 50% (1/2)] and better PFS (9.5 *vs*. 4.2 months). In order to investigate if circulating levels of miR-21 reflected expression of miR-21 in the primary tumour, we assessed miR-21 expression by In Situ Hybridization (ISH) in PDAC tissues. Matched baseline PDAC tissue was retrieved in 4 cases. Interestingly, miR-21 expression was negative in tumours of patients with low circulating miR-21, while moderate to strong miR-21 expression was detected in tumour cells of patients with high circulating miR-21 (Figure 3).

**Table 3:** miR-21 expression and efficacy data in all registered patients

	Number of events	Number of subjects	Median Survival (Months) (95% CI)	1 Year Survival (95% CI)	Hazard Ratio (95% CI)	*p*-value
***(PFS)*** **Low miR-21** **High miR-21**	6 6	6 6	12.7 (6.9 – 18.4) 3.5 (2.8 – 4.3)	66.7 (29.1 – 100) 16.7 (0 – 46.5)	1.0 4.7 (1.1 – 19.7)	0.032
***(OS)*** **Low miR-21** **High miR-21**	6 5	6 6	15.3 (7.3 – 23.3) 5.1 (0 – 10.7)	83.3 (53.5 – 100) 33.3 (0 – 70.9)	1.0 1.46 (0.41 – 5.21)	0.564

## DISCUSSION

Genetic heterogeneity is common in PDAC and limits the efficacy benefits gained from conventional anti-cancer therapies [23]. A significant proportion of patients presents with LAPC, where treatment approach is less well defined. The role of radiotherapy in management of LAPC remains a debatable issue. Induction chemotherapy is often used to better identify tumours that are already associated with micro-metastatic disease and are unlikely to benefit from localised treatments. Recent data from the LAP-07 trial suggested that CRT following NACT may improve quality of life by prolonging the time without treatment and reducing the risk of local progression, despite no impact on OS. The CirCe 07 translational ancillary study to the LAP-07 suggested that specific biomarkers, such as positivity of Circulating Tumour Cells (CTC) at baseline, could define patients with worse OS [26]. However, CTC positivity was not associated with improvement in PFS from CRT suggesting that further investigations are required in this field. A retrospective analysis of LAPC treated by CRT or CT alone suggested that molecular biomarkers, such as copy number variation of ACTN4 can better select patients who benefit from CRT over CT alone [27]; however, prospective validation of such findings remains sparse in the literature.

In this study we present data from a prospective clinical study of CRT following NACT that evaluated the role of miR-21, which is known to be associated with pancreatic carcinogenesis [22, 28]. miR-21 is known to be highly over-expressed in PDAC and has a known association with tumour aggressiveness and poor clinical outcome [29-31]. Plasma levels of miR-21 were found to be elevated in PDAC patients compared to healthy controls [32]. We have previously shown that miR-21 modulates response to Fluorouracil (FU) [33]. miR-21 was found to be an independent prognostic factor in unselected cohorts of PDAC patients undergoing GEM-based palliative or FU-based adjuvant chemotherapy [34-35]. To our knowledge, our study is the first study to demonstrate the potential prognostic role of circulating miR-21 in a prospective cohort of patients with LAPC. Despite the small numbers in our series, we were able to show that patients with low expression of miR-21 benefit more from chemotherapy with GEM-CAP and have a prolonged PFS when radiotherapy is combined to NACT. Lack of association of low miR-21 with improved OS is likely to be related to differences in subsequent palliative treatments and small sample size. PDAC shows high cellular heterogeneity and source of circulating miR-21 can either be represented by tumour cells or stromal cells. Recent evidence suggests that miR-21 within the cancer associated fibroblasts can predict response to drug treatment more than cancer cell-derived miR-21. In order to investigate the source of circulating miR-21 in our series, we assessed miR-21 expression in the matched tissues collected at the same time of the plasma collection. Although numbers are too low to draw definite conclusions, our study is a proof of concept that free plasma miR-21 reflects the expression of miR-21 in the cancer cells and therefore can be used as a surrogate for tumoural miR-21.

Our data support the notion that altered expression of plasma miR-21 may be a prognostic biomarker that may help to guide multi-modality treatment in LAPC. Acknowledging the limitations of our study in terms of small numbers, we believe that this analysis explores the role of the miR-21 in a prospective cohort of LAPC and provides further rationale for expanding biomarker-based studies in PDAC. Based on our findings, miR-21 appears to have prognostic role in patients with LAPC. Moreover, our clinical data suggest that treatment with more efficacious contemporary NACT regimens may provide enhanced systemic control prior to consideration of localised therapy options such as radiotherapy, and thus may optimise outcomes. This approach may be particularly relevant in patients with low miR-21 expression, where better outcomes can be potentially expected. Further studies to elucidate the mechanisms by which miR-21 may increase tumour sensitivity to therapies are warranted.

## PATIENTS AND METHODS

### Patients

Eligible patients had histologically confirmed unresectable LAPC and had a World Health Organisation performance status (PS) of 0-2. Patients were considered unresectable based on at least one of the three features including a) extensive peri-pancreatic lymph node involvement, b) encasement/occlusion of the superior mesenteric vein (SMV) or SMV/portal vein confluence or c) direct involvement of superior mesenteric artery, coeliac axis, inferior vena cava or aorta. Patients who received prior chemotherapy and those with CT scan-evidence of metastatic disease were not allowed. All patients were required to have adequate bone marrow, liver and renal functions as defined by the study protocol.

### Study

Pancreatic Cancer Erbitux, Radiotherapy and UFT (PERU) (ISRCTN: 65518365) was a randomised phase II study of neo-adjuvant GEM-CAP for 16 weeks in patients with LAPC. Gemcitabine 1000mg/m2 was given as an intravenous (i.v.) infusion over 30 minutes and was administered on days 1, 8 and 15 in a 28 day cycle. Capecitabine 1660mg/m2 daily (in two divided doses) was administered orally for 21 days followed by 7 days’ rest.

Following NACT, patients were randomised between: A) UFT/LV or capecitabine plus radiotherapy (RT) or B) UFT/LV or capecitabine plus cetuximab plus RT. Capecitabine 1600mg/m2/day was given concomitanlty with RT alone in arm A or with i.v. Cetuximab (400mg/m2 on day 1 and 250mg/m2 subsequently,weekly for 5 weeks) in arm B.

The aim of this study was to assess overall survival (OS) of patients receiving CRT or CRT + cetuximab after NACT. The primary endpoint of PERU study was 1-year OS; secondary end points included progression free survival (PFS), objective response rate (ORR), biomarkers of clinical benefit. The study had a Data Monitoring Committee (DMC) which monitored efficacy and safety on the study.

### Radiotherapy quality assurance

RT (45Gy in 25 fractions) was given to tumour bed including involved nodes and tumour margins, followed by a boost to gross tumour volume (GTV) of 9Gy in 5 fractions (5.4 Gy in 3 fractions was accepted as boost if normal tissue tolerance was exceeded). CT planning scan was performed between weeks 13 to 16 of NACT. Patients were treated using a 3D conformal technique; the 95% isodose was required to cover the planning target volume (PTV). GTV was defined by contrast enhanced CT and consisted of tumour and any enhancing lymph nodes > 5mm in the typical pancreatic drainage basin. No elective nodal irradiation was performed. The boost consisted of macroscopic visible disease with a 5mm margin in all directions.

### Statistical analysis

Based on the pooled GERCOR studies, the lower limit of 95% confidence interval (CI) for the 1-year survival rate of chemotherapy alone group was estimated to be 35%. A 1-year OS rate from the time of registration of ≥55% was considered acceptable (p1) and a 1-year OS rate from the time of registration of < 35% was estimated to be ruled out as unacceptable (p0). Giving 10% drop out rate, a total of 45 patients per arm (90 patients in total) were to be randomised to reach 2-sided α = 0.05 and 80% power. However, the trial was closed pre-maturely, following emergent data from LAP-07 [25] study failing to demonstrate survival advantage with the use of NACT followed by CRT. This decision was endorsed by DMC.

Intention to Treat (ITT) population and safety population were defined as all patients who got randomised to a treatment arm as they had omplete response (CR), partial response (PR) or stable disease (SD) after 12 weeks of beginning NACT and had at least one post treatment visit. Patients were analysed as randomised. OS and PFS were calculated using Kaplan Meier in association with long-rank test for the two arms in the ITT population. 1-year OS rate was displayed with 95% CIs.

### Molecular analysis

Baseline whole blood was collected in EDTA-treated tubes; cells were removed by centrifugation for 15 minutes at 1,500 x g using a refrigerated centrifuge (4°C). Supernatant was collected and centrifuged for further 10 minutes at 1,500 x g at 4C. The resulting supernatant was designated plasma and was transferred into clean RNAse free tubes and stored at −80. RNA was extracted from 200uL using the miRCURY RNA isolation kit (Exiqon, Denmark) following supplier instructions. Fixed starting volume was maintained for each sample. 2ul of RNA was then reverse transcribed and assessed for miR-21 expression (Taqman, Lifetechnologies, Paisley UK, code 000397). In the Kaplan Meier analysis, patients were divided in two groups: low miR-21 and high miR-21 according to median value. Validation by digital droplet PCR (Biorad, Berkeley, CA, USA) was performed as previously described [36]. A no template control and a negative control for each reverse transcription reaction were included in every assay and at least 10000 droplets were assessed for each sample. miR-21 expression was assessed blinded to clinical data.

miR-21 was assessed in tissue by RNA In Situ Hybridization (ISH). A locked nucleic acid (LNA) probe with complementarity to miR-21 was labeled with 5′-digoxigenin and synthesized by Exiqon (Denmark), and ISH performed as previously described [37].

Given the lack of activity of Cetuximab we have pooled the two arms together and analysed OS and PFS by above/below median miR-21 value by Cox regression to obtain 95% CIs.

## SUPPLEMENTARY MATERIAL TABLES


