# Supplementary material for: miR-21 expression and clinical outcome in locally advanced pancreatic cancer: exploratory analysis of the pancreatic cancer Erbitux, radiotherapy and UFT (PERU) trial
Source: Oncotarget. 2016 Feb 5;7(11):12672–81. doi: 10.18632/oncotarget.7208 (PMC4914313; doi:10.18632/oncotarget.7208)
Supplement: Supplementary file 1 [file oncotarget-07-12672-s001.pdf]

miR-21 expression and clinical outcome in locally advanced pancreatic cancer: exploratory analysis of the pancreatic cancer Erbitux, radiotherapy and UFT (PERU) trial

Supplementary Material

Supplementary table 1. Radiotherapy administration details of all randomised patients. *Due to inability of patient to reach the Hospital

	**RT + FLUO (n=7)**<br>**N (%)**	**RT + FLUO + CETUX (n=6)**<br>**N (%)**	**ALL**<br>**N (%)**
Number of fractions/ Gy of RT delivered			
**0/0**	1 (14.2)	0	1 (7.7)
**28/50.4**	1 (14.2)	1 (16.7)	2 (15.4)
**30/54**	5 (71.4)	5 (83.3)	10 (76.9)
Any delay in RT			
**Yes**	2 (33.3)	0	2 (16.7)
If delay Reasons			
**Toxicity**	1 (16.7)	-	1 (8.3)
**Other***	1 (16.7)	-	1 (8.3)
Any gaps in RT			
**Yes**	1 (16.7)	0	1 (8.3)
GTV (cc)			
**Mean**	74.6	45.8	60.2
**Min - Max**	22.1 – 241.1	26.3 – 62.8	22.1 – 241.1
PTV (cc)			
**Mean**	398	300	349
**Min - Max**	204 – 875	231 - 394	204 - 875
Boost (cc)			
**Mean (sd)**	220	110	166
**Min - Max**	110 - 407	0 - 171	0 - 407

Supplementary table 2: All serious adverse reactions (SAEs) reported on the study. Others included grade III ALT rise, grade III AST rise and biliary stent blockade

TOXICITY	PHASE				
	**Neo-adjuvant**	**Chemoradiotherapy**		**Adjuvant**	
		Without Cetuximab	With Cetuximab	Without Cetuximab	With Cetuximab
**Abdominal pain**	1	0	0	0	1
**Allergic reaction**	1	0	0	0	0
**Diarrhoea**	2	0	1	0	0
**Febrile Neutropenia**	0	0	0	0	2
**Nausea**	1	1	0	0	0
**Neutropenia**	0	0	0	0	1
**Non Neutropenic infection**	6	0	0	0	0
**Pneumonitis**	1	0	0	0	0
**Vomiting**	2	1	0	0	0
**Other**	3	0	0	0	0

Supplementary table 3. miR-21 expression analysis and Disease Control Rate (DCR) in all randomised patients. (Response data were missing for one patient).

	Number of events	Number of subjects	DCR after NACT (%)	p-value (exact test)
**Low miR-21**	6	6	100	0.061
**High miR-21**	2	5	40.0	
